# Macrophage LAMTOR1 Deficiency Prevents Dietary Obesity and Insulin Resistance Through Inflammation-Induced Energy Expenditure

**DOI:** 10.3389/fcell.2021.672032

**Published:** 2021-05-20

**Authors:** Lingwen Ying, Mingliang Zhang, Xiaojing Ma, Yiming Si, Xiaoya Li, Jiaorong Su, Jun Yin, Yuqian Bao

**Affiliations:** ^1^Department of Endocrinology and Metabolism, Shanghai Jiao Tong University Affiliated Sixth People’s Hospital, Shanghai Clinical Center for Diabetes, Shanghai Key Clinical Center for Metabolic Disease, Shanghai Diabetes Institute, Shanghai Key Laboratory of Diabetes Mellitus, Shanghai, China; ^2^Department of Endocrinology and Metabolism, Shanghai Eighth People’s Hospital, Shanghai, China

**Keywords:** late endosomal/lysosomal membrane adaptor p18, Kupffer cell, lipotoxicity, liver steatosis, inflammation

## Abstract

Here, we studied the metabolic function of LAMTOR1 from macrophages using LAMTOR1 macrophage-specific knockout (MKO) mice. LAMTOR1 MKO mice showed resistance to high-fat diet (HFD)-induced obesity, lipid steatosis, and glucose metabolic disorders, with elevated levels of pro-inflammatory cytokines. The energy expenditure, oxygen consumption, and CO_2_ production increased significantly in HFD-fed MKO vs. wild-type (WT) mice. HE and immunohistochemistry staining showed a remarkable CD68^+^ Kupffer cell accumulation in the liver. Additionally, flow cytometry revealed that the proportion of macrophages and monocytes increased significantly in the liver of MKO mice. Of note, these macrophages were probably derived from the bone marrow since the proportion of CD11b^+^ cells as well as the proliferative activity was also increased in the context of femoral bone marrow cells. In addition, the Kupffer cells of both WT and KO mice were double-positive for the M1 (CD86) and M2 (CD206) markers. However, the expression of both M1 and M2 macrophage-related genes was increased in the liver of HFD-fed KO mice. Murine primary hepatocytes and Kupffer cells were further isolated and incubated with oleic acid for 24 h. The glucose output of primary hepatocytes from MKO mice was not affected. However, decreased lipid tolerance was observed in LAMTOR1-deficient Kupffer cells. Overall, our results suggest that LAMTOR1 deficiency in macrophages prevents obesity and metabolic disorders via the accumulation of Kupffer cells in the liver and the consequent hyper-inflammation and increased energy expenditure. Therefore, our results provide a new perspective for macrophage-derived LAMTOR1 in the context of systemic metabolism.

## Introduction

Hepatic lipid deposition is considered one of the most important manifestations of obesity and is closely related to metabolic disorders. Studies have shown that inflammatory programs are activated early in the context of adipose expansion and subsequent liver steatosis. This is accompanied by the activation of macrophages and the increase of pro-inflammatory cytokines (Shoelson et al., 2006; Pellicoro et al., 2014; Tomita et al., 2014; Saltiel and Olefsky, 2017), suggesting a role of macrophages in the development of obesity and metabolic disorders. However, recently, scholars have also reported the protective effects of macrophages on metabolism. In fact, hepatic macrophages (Kupffer cells) can reduce hepatic lipid deposition under certain conditions (Clementi et al., 2009; Wan et al., 2014); however, the underlying mechanism remains unknown.

Our previous studies demonstrated that either systemic or adipocyte and macrophage-specific NF-κB over-activated mouse models were resistant to high-fat diet (HFD)-induced obesity, liver steatosis, and insulin resistance. Remarkable inflammation was detected, which may be related to the tumor necrosis factor-α (TNF-α)-mediated reduced p70 ribosomal protein S6 kinase (p70S6K) activity (Zhang et al., 2008; Gao et al., 2009; Tang et al., 2010). Of note, p70S6K, the downstream kinase of the mammalian target of rapamycin complex 1 (mTORC1), is considered as an important regulator of metabolism (Patti and Kahn, 2004; Um et al., 2004, 2006; Krebs et al., 2007; Le Bacquer et al., 2007; Goberdhan et al., 2016); in addition, it may be related to inflammation-mediated metabolic disorders.

LAMTOR1, also called the late endosomal/lysosomal membrane adaptor p18, is a subunit of the Ragulator Complex (composed of LAMTOR1-5). It is responsible for anchoring the Ragulator Complex onto the lysosomal membrane (de Araujo et al., 2017). Of note, the Ragulator Complex receives and transmits amino acid signals to mTORC1/p70S6K, thereby playing an important role in the regulation of cell metabolism and growth in response to nutrient availability (Sancak et al., 2010; Su et al., 2017).

Considering that LAMTOR1 is a protein upstream of mTORC1/p70S6K and that macrophages are directly related to inflammation, here, we used LAMTOR1 macrophage-specific knockout (MKO) mice to clarify the role of macrophage LAMTOR1 in inflammation and metabolism.

## Materials and Methods

### Mice

*LAMTOR1*^*flox*^ mice and *LysM-Cre* mice (C57BL/6J background) were gifts from Dr. Shengcai Lin (Xiamen University). The strategy to generate the *LAMTOR1*^*flox*^ allele is described elsewhere (Zhang et al., 2014). LAMTOR1 MKO mice were generated via the crossing of these two strains. All LAMTOR1 MKO mice used in the current study were *LAMTOR1*^*flox/flox*^ *LysM-Cre* (heterozygote) mice; the control animals used were the littermate *LAMTOR1*^*flox/flox*^ mice. Genotyping for the *LAMTOR1*^*flox*^ and wild-type *LAMTOR1* alleles was performed using the primers: 5′ TGG CGA GTA CCA GGC CAG CTG GGT TC and 5′ GAC AAA GGC TAT TGG AAC TGA CTG AAA G; the *LysM-Cre* gene was identified using the primers: 5′ CCC AGA AAT GCC AGA TTA CG and 5′ CTT GGG CTG CCA GAA TTT CTC (Supplementary Figure 1).

All mice were maintained under specific pathogen-free (SPF) conditions. Male mice aged 6–8 weeks old were used and fed with a normal chow diet (4% fat, 3.85 kcal/g, 1010010; Jiangsu Synergistic Medicine Bioengineering Co., Ltd., Jiangsu, China) or with high-fat diet (HFD; 60% fat, 5.24 kcal/g, D12492; Research Diets, Inc., New Brunswick, NJ, United States) for 12 weeks. The intraperitoneal glucose tolerance test (IPGTT) and the intraperitoneal insulin tolerance test (IPITT) were performed as previously described with some modifications (Alimujiang et al., 2020). For IPGTT, the mice were intraperitoneally injected with glucose (1.5 g/kg body weight) after overnight fasting. For IPITT, the mice had been fasted for 6 h and were injected with insulin (Humulin R, Eli Lilly and company) at a concentration of 1 U/kg body weight. Blood glucose was monitored with a glucometer (Roche, Switzerland) at regular intervals of 0, 15, 30, 60, 90, and 120 min. Afterward, the mice were sacrificed, and their sera, adipose tissues, livers, gastrointestinal tracts, skeletal muscles, and femurs were collected. All protocols and animal studies were approved by the Ethics Committee of the Shanghai Jiao Tong University Affiliated Sixth People’s Hospital.

### Food Intake and Body Temperature

When food intake was measured on the last 7 consecutive days of the 12 weeks chow diet/HFD, the mice were housed individually. A preweighed amount of food pellets of chow diet or HFD were given on the cage tops. Then, the diet consumed was measured daily, and presented as average food intake (in Kcal) during 24 h per mouse.

Body temperature was measured with a rectal thermometer (TH212, Zhongjiao Construction Instrument Technology Development Co., Ltd., Beijing, China) in the 12th week of chow diet/HFD.

### Isolation of Primary Hepatocytes and Kupffer Cells

Murine primary hepatocytes and Kupffer cells were isolated using the two-step perfusion method as previously described with some modifications (Park et al., 2017; Alimujiang et al., 2020). Mice were anesthetized and then sequentially perfused with a calcium-free solution and a collagenase IV solution until the liver was completely digested. After filtration through a 70-μm cell mesh, the filtrate was centrifuged for 2 min at 60 × g and 4°C and the supernatant was used Kupffer cells’ isolation. Briefly, the supernatant was overlaid on the surface of gradient solutions (upper layer: 4.5 mL percoll + 0.5 mL 10 × PBS + 15 mL 1 × PBS; lower layer: 6.75 mL percoll + 0.75 mL 10 × PBS + 7.5 mL 1 × PBS) and centrifuged at 800 × g (accelerate and brake at 0) for 15 min at 4°C. Kupffer cells, retained in the middle and lower layer boundaries, were then collected, diluted with equal volumes of 1 × PBS, and centrifuge at 1,000 × g for 20 min at 4°C. Finally, the supernatants were discarded and the resulting pellets were re-suspended in RPMI 1640 containing 10% fetal bovine serum (FBS) and 1% Penicillin-Streptomycin (PS). Kupffer cells were then seeded on 24-well plates and incubated at 37°C with 5% CO_2_ for 30 min; the culture medium was then replaced to remove the non-adherent sinusoidal endothelial cells.

Additionally, the initial pellet was used for the purification of primary hepatocytes. Briefly, the pellet was re-suspended in DMEM, and centrifuged at 2000 rpm at 4°C for 2 min. Cells were seeded onto 6-well or 12-well plates in M199 containing 10% FBS and 1% PS, and incubated at 37°C with 5% CO_2_ for 4–6 h. The culture medium was then replaced to remove the non-adherent cells.

### Collection of Bone Marrow Cells

The attached skin and muscle tissues around the femur were removed. Then, after rinsing with 1 × PBS, 3 times, the condyles at both ends of the femur were cut off. The bone marrow was then flushed out into a 15-mL centrifuge tube with a 20-mL syringe with a 0.65 mm medical needle filled with DMEM.

### Cell Treatment/Experimental Incubation

Primary hepatocytes were incubated with culture medium containing oleic acid (OA, O1383; Sigma Aldrich, St. Louis, MO, United States) for 24 h at a final concentration of 0, 0.4, and 0.8 mmol/L, respectively. Kupffer cells were also incubated in culture medium containing OA (final concentration of 0, 0.1, 0.2, and 0.4 mmol/L) for 24 h. The treatments were performed with 70–80% confluent cultures.

### Biochemical Measurement

The triglyceride (TG) levels of liver tissues and primary hepatocytes were measured using the Triglyceride Assay Kit (GPO-POD; Applygen Technologies Inc., Beijing, China) according to the manufacturer’s protocol. Serum alanine aminotransferase (ALT) and aspartate aminotransferase (AST) were measured with the commercial enzymatic kits (Maccura Biotechnology Co., Ltd., China) on a 7600-120 autoanalyzer (Hitachi, Tokyo, Japan).

### Histopathological Analysis

Fresh tissue specimens (liver, adipose tissue, skeletal muscle, and gastrointestinal tract) were fixed with 4% paraformaldehyde for 48 h and then embedded in paraffin. Paraffin blocks were then sectioned, and tissue sections were subjected to hematoxylin-eosin (HE) staining or to immunohistochemistry (IHC) staining using anti-CD68 (ab955; Abcam, Cambridge, United Kingdom) or anti-F4/80 (#70076; Cell signaling technology, Danvers, MA, United States) antibodies and the standard methods (Mahoney et al., 2015). Images were captured using a light microscope (Leica Microsystems, Wetzlar, Germany). The adipocyte size, as well as the percentage of CD68- or F4/80-positive area (%Area) in the context of immunohistochemistry stained sections was quantitated using ImageJ (FIJI version; NIH, Bethesda, MD, United States) (Schindelin et al., 2012).

### Oil Red O Staining

To assess hepatic steatosis, fresh liver tissues were embedded in optimal cutting temperature compound (OCT), immediately frozen in liquid nitrogen, and sectioned. Frozen liver sections were stained with oil red O (O0625; Sigma Aldrich, St. Louis, MO, United States) according to the manufacturer’s instructions. For *in vitro* studies, primary hepatocytes and Kupffer cells were washed with 1 × PBS twice and fixed in 4% paraformaldehyde for 10 min. Fixed cells were stained with oil red O diluent (saturated oil red O solution and distilled water in a 3:2 ratio) for 15 min, washed with 60% ethanol and then with 1 × PBS. After 10 min of nuclear counterstaining with hematoxylin, preparations were washed once with water and observed under a light microscope (Leica Microsystems, Wetzlar, Germany).

### Gluconeogenesis Analysis

After primary hepatocytes adhered to the plates, the culture medium was replaced with serum-free medium (M199 supplemented with 1% BSA and 1% PS) and cells were starved overnight. Glucose-producing solution, i.e., phenol red-free, glucose-free DMEM, containing substrates needed for gluconeogenesis (20 mmol/L lactate, 2 mmol/L pyruvate, 2 mmol/L L-glutamine, and 15 mmol/L HEPES) was prepared. Then, primary hepatocytes were incubated at 37°C for 6 h with a glucose-producing solution with, or without 0.1 mmol/L pCPT-cAMP (Sigma Aldrich, St. Louis, MO, United States) and 1 μmol/L dexamethasone (Sigma Aldrich). Two-hundred microliter of the culture medium were then collected, and the glucose concentration was measured using a colorimetric assay kit (glucose oxidase method; E1010; Applygen, Beijing, China).

### Quantitative Real-Time Polymerase Chain Reaction (qRT-PCR)

Total mRNAs from frozen livers and adipose tissues were extracted using the TRIZOL method, and cDNA was synthesized using the HiScript^®^ II Reverse Transcriptase System (R223-01; Vazyme, Nanjing, China). The real-time quantitative PCR system LightCycler^®^ 480 II (Roche, Hercules, CA, United States) was used for quantitative analysis. All procedures were performed according to the manufacturers’ protocols. The mRNA expression of different genes was normalized to that of β*-actin* or *GAPDH*. The sequences of the primers used for qRT-PCR are listed in Supplementary Table 1.

### Enzyme-Linked Immunosorbent Assay (ELISA)

The concentrations of interleukin 1β (IL-1β; ZL2040-A; R&D systems, Minneapolis, MN, United States), IL-6 (ZL2163-A; R&D systems), nitric oxide synthase 2 (NOS2; ZL30326-A; R&D systems), TNF-α (ZL2132-A; R&D systems), and C-C motif chemokine ligand 2 (CCL2; ZL2720-A; R&D systems) in serum samples and Kupffer cell supernatants were measured by ELISA using the abovementioned commercial kits, according to the manufacturer’s instructions.

### Western Blot Analysis

Total proteins were extracted from liver tissues, primary hepatocytes, and Kupffer cells using RIPA buffer (P0013B Beyotime, Jiangsu, China). Protein samples (20–30 μg) were separated using SDS-PAGE (10–15%; Bio-Rad, Hercules, CA, United States) and transferred onto PVDF membranes (Millipore Corporation, Burlington, MA, United States). After blocking with 5% skimmed milk, the membranes were incubated with primary antibodies overnight at 4°C, and then incubated with horseradish peroxidase-conjugated secondary antibodies for 1 h at room temperature (22–25°C). Antibodies against LAMTOR1 (8975S), mTOR (2983S), p-mTOR (2971S), p70S6K (2708S), p-p70S6K (9234S), and β-actin (3700S), as well as anti-mouse (7076) and anti-rabbit (7074) secondary antibodies were all purchased from Cell Signaling Technology (Danvers, MA, United States). Signals were detected using the ImageQuant LAS 4000 mini system (GE Healthcare, Chicago, IL, United States).

### Flow Cytometry

In order to clarify the proliferation activity of the mononuclear phagocytic system (MPS) of the bone marrow, femoral bone marrow cells were isolated and stained with anti-D11b (25-0112-81; eBioscience, San Diego, CA, United States), anti-CD45 (35-0451-80; eBioscience), and anti-Ki67 (17-5698-80; eBioscience) antibodies. To disclose the ratio of Kupffer cells and hepatocytes, the cell suspensions obtained via the two-step perfusion method (containing both Kupffer cells and hepatocytes) were stained with anti-CK18 (ab181597 and ab6717; Abcam, Cambridge, United Kingdom), anti-CD11b, F4/80 (123131; Biolegend, San Diego, CA, United States), and anti-CD45 (35-0451-80; eBioscience) antibodies, together with a viability dye (65-0866-18; eBioscience). Cells were acquired on the BD FACSCanto^TM^ II system (BD Biosciences, Franklin Lakes, NJ, United States). The appropriate FSC- and SSC-based gating strategy was used to analyze each population.

### Detection of Cell Apoptosis Using the Cell Counting Kit 8 (CCK8)

Kupffer cell apoptosis after OA treatment was evaluated using the cell counting kit 8 (CCK8; Dojindo Molecular Technologies, Rockville, MD, United States) according to the manufacturer’s protocol. Briefly, Kupffer cells were seeded into 96-well plates at 5 × 10^3^ cells/well in quadruplicate and treated with 0, 0.1, 0.2 and 0.4 mmol/L OA for 24 h. The CCK-8 solution (10 μL) was then added to each well and the cells were cultured for 2–2.5 h in a humidified atmosphere of 95% air and 5% CO_2_ at 37°C. A microplate reader (SpectraMax i3; Molecular Devices LLC., San Jose, CA, United States) was used to measure the absorbance at 450 nm.

### 3-(4,5-Dimethylthiazol-2-yl)-2,5-Diphenyltetrazolium Bromide (MTT) Cell Viability Assay

Cell viability of primary hepatocytes and Kupffer cells after OA treatment was evaluated using the MTT kit (C0009S, Beyotime, Jiangsu, China) according to the manufacturer’s protocol. Briefly, primary hepatocytes and Kupffer cells were seeded into 96-well plates at 5 × 10^3^ cells/well in quadruplicate and treated with 0, 0.1, 0.2, 0.4, 0.6, 0.8, 1.0, 1.2, and 1.5 mmol/L OA for 24 h. The MTT solution (10 μL) was then added to each well and the cells were cultured for 4 h in a humidified atmosphere of 95% air and 5% CO_2_ at 37°C. Subsequently, formazan solvent (100 μL) was added to each well and the cells were cultured for another 4 h until all the formazan dissolved. A microplate reader (SpectraMax i3; Molecular Devices LLC., San Jose, CA, United States) was used to measure the absorbance at 570 nm.

### Metabolic Cages

Indirect calorimetry measurements were performed using the High-definition multiplexed respirometry system (Sable Promethion; Sable Systems International, North Las Vegas, NV, United States). Each individual mouse was placed in a chamber for 3 consecutive days at 23.0°C with 12 h light/dark cycles. After 24 h (acclimation period), data including the oxygen consumption (VO_2_), carbon dioxide production (VCO_2_), and respiratory exchange ratio (RER) were recorded every 5 min. The energy expenditure (kcal/kg/h) was calculated as previously reported (Tang et al., 2010).

### Statistical Analysis

All parameters are presented as the mean ± standard error of the mean (SEM). Statistical differences of intergroup comparison were evaluated using the Student’s *t*-test (two groups) or ANOVA with SNK method (multiple groups). A two-tailed *P-*value < 0.05 was considered statistically significant.

## Results

### The Phosphorylation of mTOR and S6K Is Decreased in LAMTOR1-Depleted Macrophages

**FIGURE 1:**
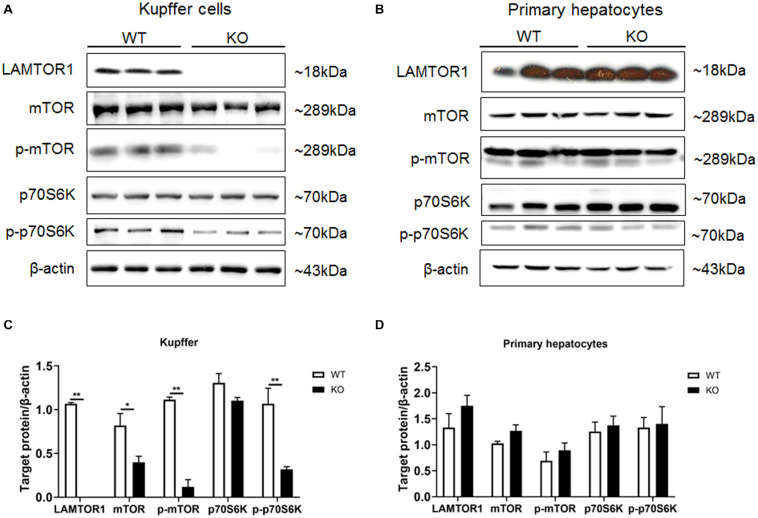
LAMTOR1 MKO mice were successfully generated. **(A,B)** Protein expression of LAMTOR1/mTORC1/p70S6K in Kupffer cells **(A)** and primary hepatocytes **(B)** analyzed by western blotting. **(C,D)** Quantification of the protein expression of LAMTOR1/mTORC1/p70S6K in Kupffer cells **(C)** and primary hepatocytes **(D)**. *n* = 4–6. *P < 0.05 vs. WT; **P < 0.01 vs. WT. KO, myeloid-specific knockout mice; WT, wild-type.

Primary hepatocytes and Kupffer cells of LAMTOR1 MKO and wild-type (WT) mice were isolated and analyzed. Western blot analysis showed that LAMTOR1 was expressed in hepatocytes but not in Kupffer cells, suggesting that the MKO model was successfully constructed (Figure 1). Of note, the LAMTOR1 MKO mice appeared healthy and presented a normal breeding pattern since both male and female homozygous mutant animals were fertile and healthy offsprings were obtained through breeding of KO x KO.

Next, the expression and phosphorylation of the downstream mTOR and S6K were investigated. The results showed the downregulation of the expression of mTOR, but almost unchanged protein levels of p70S6K in Kupffer cells. However, the phosphorylation of mTOR and p70S6K was significantly reduced in KO mice (Figure 1).

### LAMTOR1 MKO Mice Are Lean and Resistant to HFD-Induced Glucose Metabolic Disorders

**FIGURE 2:**
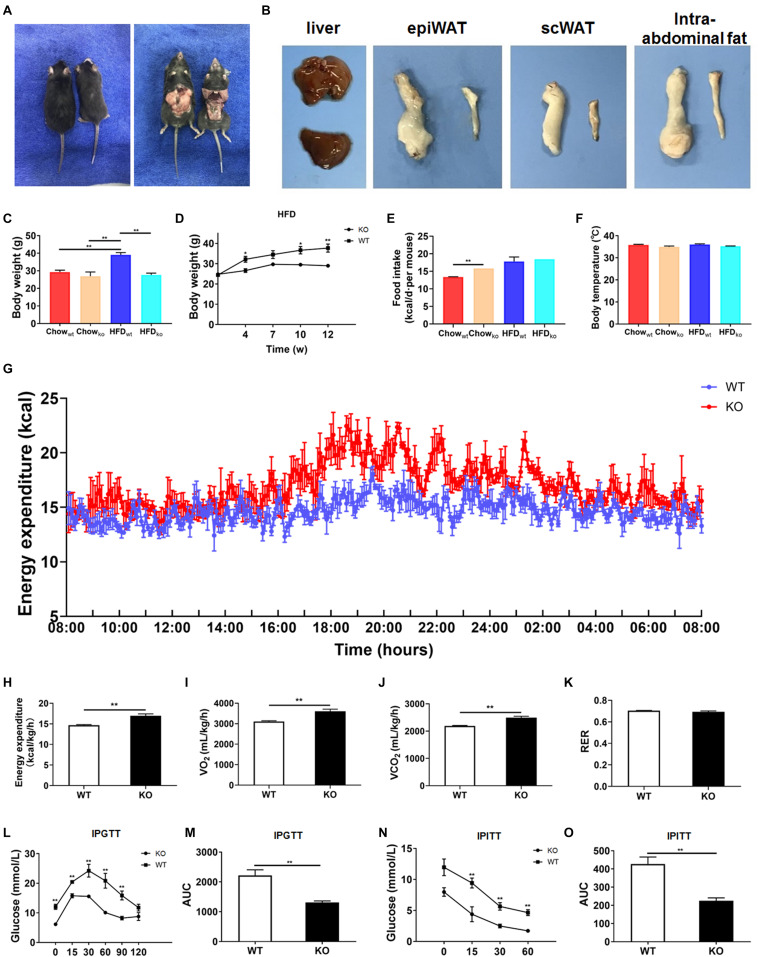
LAMTOR1 MKO mice are resistant to dietary-induced obesity and glucose metabolism disorders. **(A)** Representative photos of HFD-fed WT (left) and LAMTOR1 MKO (right) mice. **(B)** Representative images of the liver and white adipose tissue (epiWAT, scWAT, and intra-abdominal fat) of HFD-fed WT (upper or left) and LAMTOR1 MKO (below or left) mice. **(C)** Body weight after 3 months of intake of Chow diet or HFD. **(D)** Body weight variation during the intake of HFD. **(E)** Food intake. **(F)** Body temperature. (G-K) Metabolic cages-related results of **(G,H)** energy expenditure, **(I)** oxygen consumption, **(J)** CO_2_ production, and **(K)** RER. The test was conducted at 12 weeks of age with HFD, and prior to metabolic cage test, the mice had been fed with HFD for 4 weeks. (**L–O)** IPGTT (glucose 1.5 g/kg) and IPITT (insulin 1 U/kg) were performed after 3 months of HFD intake. The data expressed as the mean ± SEM, *n* = 4–6. **P < 0.01 vs. WT. epiWAT, epididymal white adipose tissue; HFD, high-fat diet; IPGTT, intraperitoneal glucose tolerance test; IPITT, intraperitoneal insulin tolerance test; MKO, myeloid-specific knockout; RER, respiratory exchange ratio; scWAT, subcutaneous white adipose tissue; SEM, standard error of the mean; WT, wild-type.

Next, we comprehensively investigated the phenotype of LAMTOR1 MKO mice. There was no significant difference between MKO and WT mice fed with chow diet in terms of metabolic parameters (e.g., glucose and insulin tolerance; Supplementary Figure 2). However, when fed with an HFD, LAMTOR1 MKO mice showed a significant decrease in the body weight and white adipose tissue (WAT) mass, as well as a smaller liver (vs. WT mice), suggesting the macrophage-specific depletion of LAMTOR1 was able to prevent HFD-induced obesity (Figures 2A–D).

In order to investigate the mechanism behind the reduction in body weight in MKO vs. WT mice in the context of HFD, the food intake, body temperature, and energy expenditure were measured. When fed with a chow diet, LAMTOR1 MKO mice showed a higher food intake; on the other hand, the food intake of both MKO and WT mice was equivalent in the context of HFD (Figure 2E). In addition, the body temperature was also stable at 35.5 ± 0.6°C (Figure 2F). Interestingly, the HFD-fed MKO mice showed an elevation in energy expenditure, as well as oxygen consumption and CO_2_ production, while the RER remained equivalent to that in HFD-fed WT mice (Figures 2G–K). Overall, these results suggest that the decreased weight of LAMTOR1 MKO mice fed an HFD was due to an increase in energy expenditure, rather than due to suppressed appetite or increased heat production.

We also determined the effect of LAMTOR1 MKO on glucose metabolism. The IPGTT results demonstrated that the blood glucose levels of HFD-fed MKO mice at 0, 15, 30, 60, and 90 min were significantly lower compared to those of WT mice (Figures 2L,M). Consistent with the IPGTT results, we found that the glucose levels of MKO mice were much lower than those in the control group at 15, 30, and 60 min of IPITT (IPITT was stopped 60 min after insulin injection because the glucose levels of LAMTOR1 MKO mice declined to 1.4–2.1 mmol/L; Figures 2N,O). Altogether, the above results suggest that the macrophage-specific depletion of LAMTOR1 prevents HFD-induced insulin resistance.

### Macrophage Infiltration Is Not Impacted in the WAT, Muscles, and Gut of LAMTOR1 MKO

**FIGURE 3:**
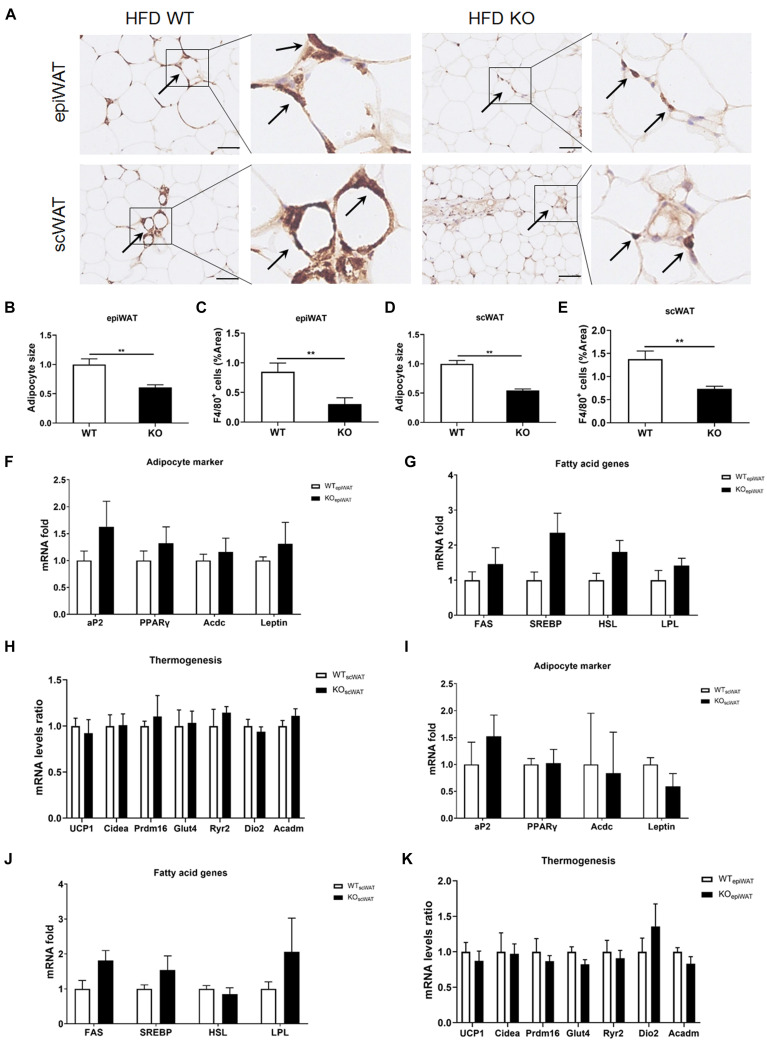
LAMTOR1 depletion in macrophages reduces WAT mass. **(A)** IHC staining (F4/80) in the epiWAT and scWAT of HFD-fed mice. Black arrow represents the F4/80^+^ macrophages. **(B–E)** Quantification of adipocyte size and the F4/80^+^ macrophage/%area in the epiWAT and scWAT of HFD-fed mice. The IHC quantification is performed in a total of 36 images, with 3 images per mouse, and is presented as the percentage of the area of F4/80^+^ cells. **(F–K)** mRNA expression of adipocyte-specific genes, fatty acid genes (including both fat synthesis- and lipolysis-related genes) and thermogenesis in the epiWAT **(F–H)** and scWAT **(I–K)** of HFD-fed WT and LAMTOR1 MKO mice. The data are expressed as the mean ± SEM, *n* = 4–6. **P < 0.01 vs. WT. epiWAT, epididymal white adipose tissue; HFD, high-fat diet; IHC, Immunohistochemistry; MKO, myeloid-specific knockout; scWAT, subcutaneous white adipose tissue; SEM, standard error of the mean; WT, wild-type.

Next, we evaluated macrophage infiltration in various tissues. In line with the lean phenotype of LAMTOR1 MKO mice in the context of HFD, first, we focused on the WAT. Compared with WT mice, the size of adipocytes in both subcutaneous (scWAT) and epididymal WAT (epiWAT) of MKO mice decreased nearly 50%. Meanwhile, there was a significant reduction in the levels of F4/80^+^ macrophages in both epiWAT and scWAT of HFD-fed LAMTOR1 MKO mice as per IHC staining (Figures 3A–E). However, the expression of adipocyte markers such as adipocyte Protein 2 (*aP2*), peroxisome proliferator-activated receptors γ (*PPAR*γ), adiponectin (*Acdc*) and leptin, as well as of fatty acid synthesis- and lipolysis-related genes including fatty acid synthase (*FAS*), sterol response element-binding protein (*SREBP*), hormone-sensitive lipase (*HSL*), and lipoprotein lipase (*LPL*), did not differ between WT and MKO mice. Similarly, no difference was observed in expression of genes related to thermogenesis, e.g., uncoupling protein (*UCP-1*), cell death-inducing DNA fragmentation factor alpha-like effector A (*Cidea*), PR/SET Domain 16 (*Prdm16*), glucose transporter 4 (*Glut4*), Ryanodine Receptor 2 (*Ryr2*) and iodothyronine deiodinase 2 (*Dio2*), between WT and MKO mice (Figures 3F–K). It suggested that WAT, though played a role, may not be the initial factor of the metabolic improvement observed in LAMTOR1 MKO mice.

The skeletal muscles are the tissues with higher energy expenditure, and the gut plays a pivotal role in energy intake. Thus, we wondered whether LAMTOR1 MKO induced skeletal muscle or gastrointestinal changes. Curiously, we found no differences in the general appearance, HE staining, or IHC staining (Supplementary Figure 3) in the skeletal (gastrocnemius and soleus) muscles from HFD-fed LAMTOR1 MKO and littermate control (WT) mice. Similarly, the general appearance of the gastrointestinal tract (including the stomach, duodenum, jejunum, ileum, cecum, and colon) of KO mice was normal. Of note, HE staining and F4/80 IHC staining showed no changes in the gastrointestinal mucosal structure and macrophage infiltration, respectively (Supplementary Figure 4).

### LAMTOR1 MKO Leads to the Accumulation of Macrophages in the Liver

**FIGURE 4:**
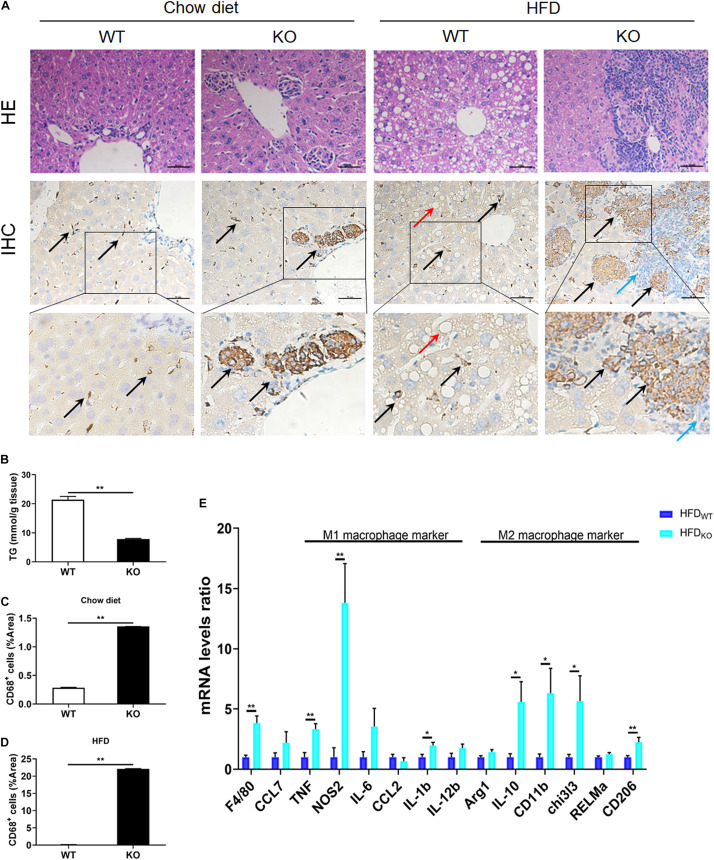
LAMTOR1 MKO attenuates hepatic steatosis and leads to accumulation of macrophages in the central vein area and the portal area. **(A)** HE and CD68 IHC staining in the liver. Black arrow represents the CD68^+^ Kupffer cells, red arrow represents the lipid droplets, and blue arrow represents the dead cells. **(B)** Hepatic TG concentration of HFD-fed WT and KO mice. **(C,D)** IHC quantification in the context of **(C)** Chow diet- and **(D)** HFD-fed mice, presented as the percentage of the area of CD68^+^ cells. **(E)** qRT-PCR results of the expression of inflammatory genes. The data expressed as mean ± SEM, *n* = 4–6. *P < 0.05 vs. WT; **P < 0.01 vs. WT. HE, hematoxylin & eosin staining; HFD, high-fat diet; IHC, Immunohistochemistry; MKO, myeloid-specific knockout; SEM, standard error of the mean; WT, wild-type.

The liver serves as the center of glycolipid metabolism. Therefore, we performed liver HE staining and found a large number of lipid deposits in the livers of WT mice after 3 months of HFD intake, while the KO mice showed no hepatic steatosis, as well as a significantly reduced hepatic TG concentration (Figures 4A,B and Supplementary Figure 5). In addition, there were inflammatory foci in both chow diet- and HFD-fed MKO mice, suggesting the spontaneous hepatic aggregation/accumulation of immune cells. Of note, more severe inflammatory foci were observed in the livers of HFD-fed MKO mice. In addition, these inflammatory foci mainly located around the epithelial cells in the central vein area, as well as the portal area. IHC staining revealed that half of the inflammatory foci were dead cells and CD68^+^ Kupffer cells were the main component of the living cells in these foci (Figures 4A–D).

Since macrophages were the main cells in liver infiltrates, next we measured the expression of M1 and M2 macrophage-related genes in the liver. The results showed a simultaneous increase in the expression of M1 and M2 macrophage-related genes in HFD-fed MKO mice (Figure 4E), suggesting a synergistic increase in the activity of M1/M2 Kupffer cells. Therefore, it is reasonable to conclude that the liver may be responsible for the metabolism improvement in LAMTOR1 MKO.

### Monocytes and Macrophages Are Recruited Into the Liver in LAMTOR1 MKO Mice

**FIGURE 5:**
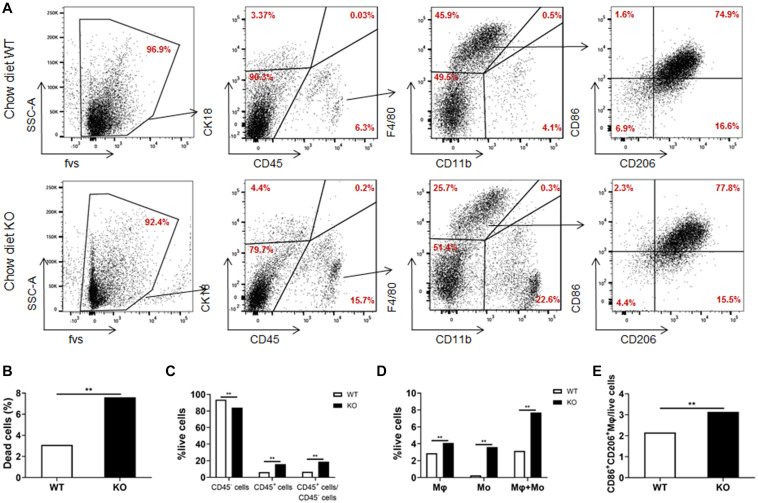
Increased apoptosis and aggregation of immune cells in the liver of LAMTOR1 MKO mice. **(A)** Flow cytometry results (presented with same cell amount). **(B)** Percentage of dead cells. **(C)** Percentage of CD45^–^ cells, CD45^+^ cells, and the ratio of CD45^+^/CD45^–^ cells within live cells. **(D)** Percentage of macrophages (F4/80^+^CD11b^–^), monocytes (F4/80^–^CD11b^+^), and sum of macrophages and monocytes within live cells. **(E)** Percentage of M1- and M2-like macrophages in live cells. *n* = 4. **P < 0.01 vs. WT. KO, myeloid-specific knockout mice; Mo, monocytes; Mφ, macrophages; WT, wild-type.

We further analyzed the cell composition of the liver. Since it was difficult to obtain high-quality primary hepatocytes from HFD-fed obese mice, livers from chow diet-fed LAMTOR1 MKO and WT mice were used for flow cytometry analysis. The results showed that the dead cells in MKO mice were about 2.5 times higher than those in WT mice (Figures 5A,B). As for the living cells, compared with control mice, the proportion of CD45^+^ immune cells increased significantly in LAMTOR1 MKO mice (15.9% vs. 6.3%, *P* < 0.01; Figures 5A,C). In parallel, a significant decrease in the proportion of CD45^–^ cells (mainly comprised of primary hepatocytes, and a few non-parenchymal cells such as vascular endothelial cells) was detected in MKO vs. WT mice (84.1% vs. 93.7%, *P* < 0.01), resulting in a CD45^+^/CD45^–^ cell ratio of up to 18.95% in LAMTOR1 MKO mice (Figures 5A,C). Importantly, consistent with the results of HE and IHC staining, flow cytometry confirmed the accumulation of immune cells in the liver of LAMTOR1 MKO mice.

In line with the above results, we also found that the frequency of macrophages and monocytes within live cells was higher in MKO vs. WT mice (macrophages: 4.1% vs. 2.9%; monocytes: 3.6% vs. 0.3%, both *P* < 0.01), resulting in a remarkably higher sum of macrophages and monocytes in MKO vs. WT mice (7.7% vs. 3.2%, *P* < 0.01; Figures 5A,D). Of note, we also found that the proportion of CD86^+^CD206^+^ cells within live cells increased remarkably in MKO mice (3.1% vs. 2.2%, *P* < 0.01; Figure 5E), in line with the increase in the expression of M1 and M2 macrophage-related genes in the liver. Thus, we hypothesized that the depletion of LAMTOR1 in macrophages made Kupffer cells more prone to apoptosis, leading to the consequent increased recruitment of monocytes to the liver and transformation into Kupffer cells to maintain liver homeostasis.

### The Bone Marrow Is the Main Source of Macrophages Accumulated in the Liver of LAMTOR1 MKO Mice

**FIGURE 6:**
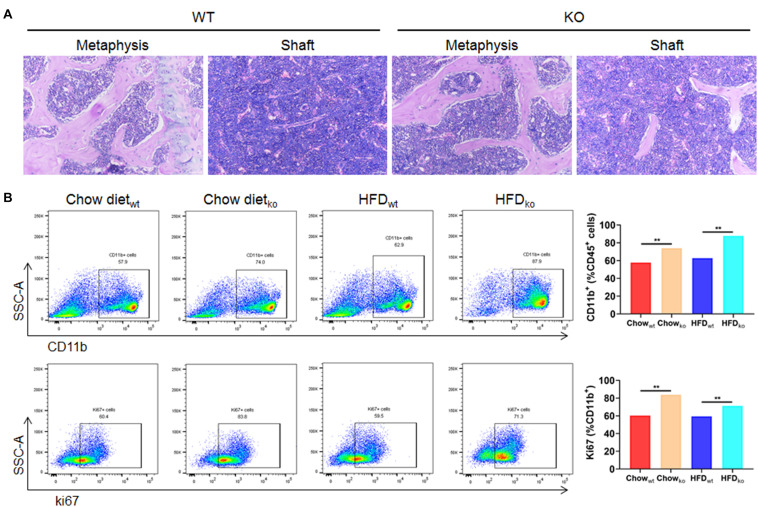
The proliferation of cells from the bone marrow mononuclear phagocyte system is increased in LAMTOR1 MKO mice. **(A)** HE staining of the bone marrow. **(B)** Flow cytometry analysis of bone marrow cells. The results refer to the bone marrow of the left femurs since both left and right femoral bone marrow displayed the same results. *n* = 4. **P < 0.01 vs. WT. HFD, high-fat diet; KO, myeloid-specific knockout mice; WT, wild-type.

In order to clarify where the monocytes and macrophages were recruited to the liver, murine femoral bone marrow was collected and analyzed via HE staining and flow cytometry. The results showed that the proportion of CD45^+^CD11b^+^ cells (mainly monocytes and macrophage) in LAMTOR1 MKO mice increased about 28 and 40% compared to that of WT mice fed with chow diet and HFD, respectively (Figures 6A,B). In addition, the corresponding proliferative activity (assessed by Ki67^+^) also increased significantly compared to that in the context of WT mice (Figure 6B). Altogether, these data suggest that macrophage LAMTOR1 deficiency induces the proliferation of cells of the MPS in the bone marrow, the main source of the macrophages/monocytes infiltrating into the liver.

### LAMTOR1 Deficiency Leads to the Decreased Lipid Tolerance in Kupffer Cells

**FIGURE 7:**
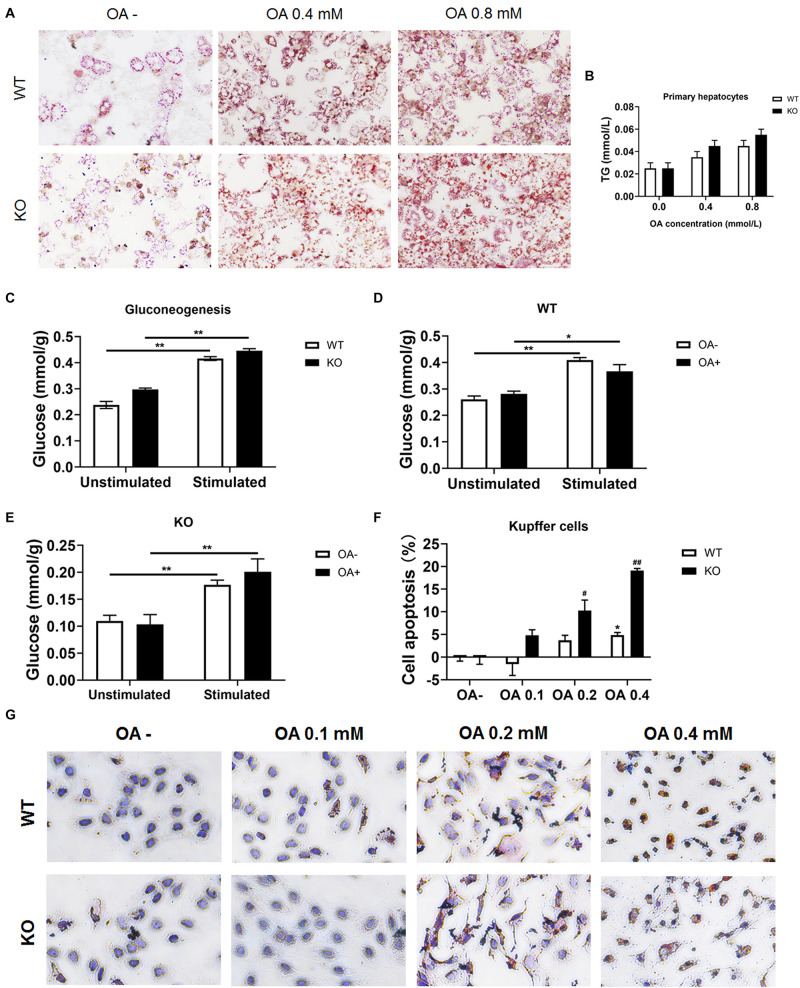
LAMTOR1 deficiency reduces lipid tolerance of Kupffer cells. **(A)** Red O staining of murine primary hepatocytes after incubation with 0, 0.4, and 0.8 mmol/L OA for 24 h. **(B)** Intracellular TG levels in primary hepatocytes after OA (0, 0.4, and 0.8 mmol/L) treatment. **(C)** Gluconeogenesis in the primary hepatocytes of WT and KO mice. **(D,E)** Gluconeogenesis in the primary hepatocytes of WT **(D)** and KO **(E)** mice in the presence of 0 or 0.8 mmol/L OA for 24 h. **(F)** CCK8 results in the context of Kupffer cells incubated with 0, 0.1, 0.2, and 0.4 mmol/L OA for 24 h (presented as cell apoptosis%). **(G)** Red O staining of murine Kupffer cells after incubation with 0, 0.1, 0.2, and 0.4 mmol/L OA for 24 h. The data expressed as the mean ± SEM. *P < 0.05 vs. WT **(C–E)** or WT OA- **(F)**; **P < 0.01 vs. WT **(C–E)** or WT OA- **(F)**; ^#^*P* < 0.05 vs. KO OA-; ^##^*P* < 0.01 vs. KO OA-. KO, myeloid-specific knockout mice; OA, oleic acid; WT, wild-type.

To investigate whether hepatocytes function was affected, serum ALT and AST were determined. We found both ALT and AST showed no difference between chow diet-fed WT and KO mice. As for the HFD-fed mice, considering the improved hepatic lipid deposition, the KO mice showed a significantly lower ALT and AST levels compared with the WT mice (both *P* < 0.05; Supplementary Figures 6A–D). Moreover, primary hepatocytes were isolated and incubated with OA for 24 h, cellular viability were measured by MTT test (Supplementary Figures 6E,F) and lipid deposition was evaluated via oil red O staining. The lipid deposition increased with the increased concentration of OA in the culture medium; the triglycerides also increased in a dose-dependent manner (Figures 7A,B). In addition, the glucose output of primary hepatocytes of MKO mice was comparable to that of control mice both in the absence and presence of OA (Figures 7C–E), suggesting the LAMTOR1 MKO does not affect hepatocytes.

Kupffer cells were also isolated and incubated with 0, 0.1, 0.2, and 0.4 mmol/L OA for 24 h. The results showed that Kupffer cells of control mice could only tolerate 0.2 mmol/L or less of OA since the apoptosis rate increased significantly (up to 7.3%) in the context of 0.4 mmol/L OA. On the other hand, Kupffer cells of LAMTOR1 MKO mice were only able to survive well in the presence of 0.1 mmol/L OA, with apoptosis rates of up to 10.3 and 19.1%, under 0.2 and 0.4 mmol/L OA, respectively (Figures 7F,G and Supplementary Figures 6G,H). Altogether, these above results support the hypothesis that the increased apoptosis of Kupffer cells in the liver of MKO mice is due to their decreased tolerance to lipids.

### LAMTOR1 MKO Mice Show an Hyper-Inflammatory State

**FIGURE 8:**
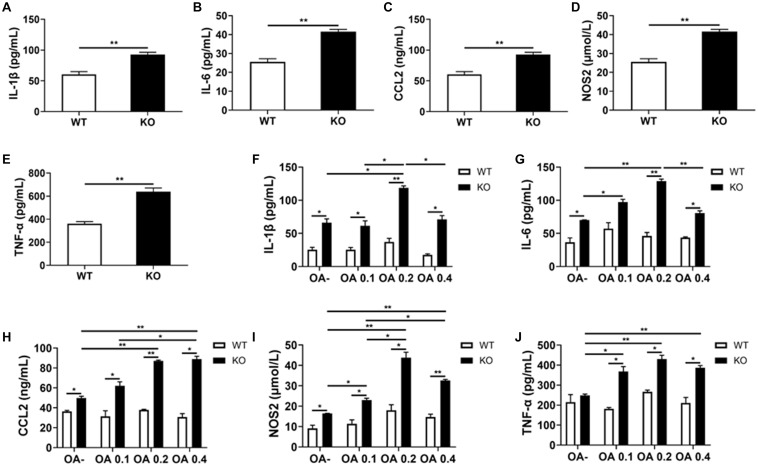
LAMTOR1 MKO increases the levels of inflammatory factors. **(A–E)** Serum levels of inflammatory cytokines and of the CCL2 chemokine in HFD-fed mice. **(F–J)** Secretion of inflammatory cytokines and of the CCL2 chemokine in the context of Kupffer cells incubated with 0, 0.1, 0.2, and 0.4 mmol/L OA for 24 h. All of these inflammatory factors were measured via ELISA. The data expressed as the mean ± SEM, *n* = 4–6. *P < 0.05; **P < 0.01. CCL2, C-C motif chemokine ligand 2; IL, Interleukin; KO, myeloid-specific knockout mice; NOS2, nitric oxide synthase 2; OA, oleic acid; TNF-α, tumor necrosis factor α; WT, wild-type.

Consistent with the former RT-PCR results, we detected a significant increase in the serum levels of IL-1β, IL-6, CCL2, NOS2, and TNF-α in HFD-fed MKO mice (as per ELISA), indicating hyper-inflammation (Figures 8A–E). In addition, the supernatants of Kupffer cells incubated with 0, 0.1, 0.2, and 0.4 mmol/L OA for 24 h also showed increased levels of IL-1β, IL-6, CCL2, NOS2, and TNF-α. Of note, these levels were already increased at baseline in the context of MKO vs. WT mice, and further increased in the presence of OA, peaking at 0.2 mmol/L (0.6–2.2 times higher than the levels in the context of WT cells; Figures 8F–J). Altogether, the above results suggest that the hyper-inflammatory state in LAMTOR1 MKO mice is probably due to the overactivation of Kupffer cells.

## Discussion

**FIGURE 9:**
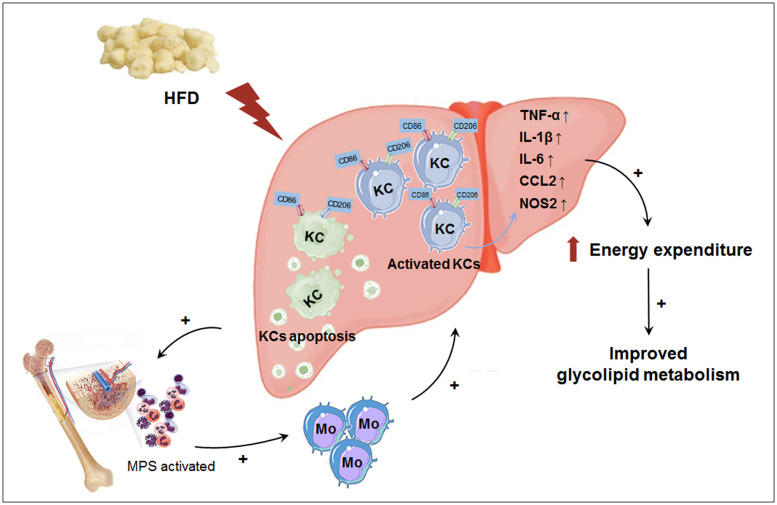
Macrophage LAMTOR1 deficiency prevents dietary obesity and insulin resistance through inflammation-induced energy expenditure. Kupffer cells presented as CD86^+^ CD206^+^. Macrophage LAMTOR1 deficiency led to a spontaneous hepatic aggregation/accumulation of Kupffer cells. While fed with HFD, more LAMTOR1-null Kupffer cells located around the epithelial cells in the central vein area, as well as the portal area because of the decreased lipid tolerance. The bone marrow, with enhanced proliferation of MPS induced by apoptosis of Kupffer cells, was the main source of macrophages accumulated in the liver. Meanwhile, overactivated Kupffer cells induced hyperinflammatory state, indicated by increased cytokines including TNF-α, IL-1β, and IL-6, resulted in elevated energy expenditure and improved glycolipid metabolism. CCL2, C-C motif chemokine ligand 2; HFD, high-fat diet; IL, Interleukin; KC, Kupffer cell; KO, myeloid-specific knockout mice; Mo, monocyte; MPS, mononuclear phagocytic system; NOS2, nitric oxide synthase 2; TNF-α, tumor necrosis factor α; WT, wild-type.

In this study, we explored the effect of macrophage LAMTOR1 on diet-induced obesity and glycolipid metabolism. We found that LAMTOR1 MKO mice were resistant to obesity and metabolic disorders with an elevation in energy expenditure. Interestingly, the liver showed a spontaneous accumulation of Kupffer cells and monocytes after macrophage LAMTOR1 depletion (Figure 9).

In previous studies based on LAMTOR1 conditional knockout models, the role of LAMTOR1 in metabolism was rarely explored. Only one recent study reported that the β-cell-specific knockout of LAMTOR1 resulted in improved glucose tolerance and increased glucose-stimulated insulin secretion (Huang et al., 2020). For the first time, this study explored the role of macrophage LAMTOR1 in metabolism. Our results show that the specific depletion of LAMTOR1 in macrophages is beneficial for glycolipid and energy metabolism.

LAMTOR1 is upstream of mTORC1. Previous studies have shown that mTORC1 serves as a sensor to regulate nutrients (amino acids and glucose) and insulin signals (Patti and Kahn, 2004; Um et al., 2006). Jiang et al. (2014) generated mice deficient in the regulatory associated protein of mTORC1 (Raptor) in macrophages and found that macrophage Raptor deficiency resulted in improved systemic insulin sensitivity, in line with our results. However, HFD-fed Raptor MKO mice showed reduced inflammatory gene expression, as well as reduced numbers of macrophages in liver and adipose tissues without impact on the body weight. In contrast, in the current study, LAMTOR1 MKO mice were lean and showed a hyper-inflammatory status, along with a spontaneous accumulation of macrophages in the liver. The differences in the body weight of LAMTOR1 and mTORC1 MKO mice may be related to the different status of inflammation and energy metabolism. Therefore, the respective role of LAMTOR1 and mTORC1 in the regulation of inflammation requires further investigation.

Several studies have reported the relationship between inflammation and energy expenditure. The concentration of inflammatory cytokines such as TNF-α, IL-1, and IL-6 positively correlated with energy expenditure (Ye and McGuinness, 2013). Accordingly, TNF-α, IL-1, or IL-6-depleted mice showed higher body weight (Wallenius et al., 2002; Pamir et al., 2009). Of note, the TNF-α-enhanced leptin activity may also play a role in the context of elevated energy expenditure (Gan et al., 2012). Furthermore, the central administration of IL-6, as well as the overexpression of IL-6 in the central nervous system, resulted in increased energy expenditure and decreased body weight gain (Timper et al., 2017). Importantly, the above data are consistent with those produced in the context of pharmacological studies. Anti-inflammatory medicines including salsalate and celecoxib, as well as anti-TNF-α and anti-IL-6 antibodies, induced weight gain and fat accumulation, with declined energy expenditure, supporting the role of inflammation in energy metabolism (Wang and Ye, 2015). Consistent with all of these studies, our data showed increased TNF-α, IL-1, and IL-6 systemic levels, elevated energy expenditure, and lower body weight in LAMTOR1 MKO mice fed with HFD.

Except for the systematic hyper-inflammation and improvement of metabolic disorders, we also found the aggregation of Kupffer cells in the livers of LAMTOR1 MKO mice. Kupffer cells account for the largest number of tissue-resident macrophages in the body (80–90%) and play an important role in the maintenance of liver (and systemic) homeostasis (Guilliams et al., 2016; Krenkel and Tacke, 2017). Our study further suggests a pivotal role of Kupffer cells in the context of systemic metabolism.

Generally, macrophages are categorized as classically (M1) and alternatively (M2) activated. M1 macrophages are involved in the triggering of intense inflammation and tissue damage (Mantovani et al., 2013), while M2 macrophages are associated with T helper 2 cell-mediated responses, tissue repair, and fibrosis (Gundra et al., 2014; Stifano and Christmann, 2016). Kimura et al. (2016) found that macrophage-specific LAMTOR1 deficiency resulted in abnormally enhanced M1 and defective M2 *in vitro* polarization in the presence of lipopolysaccharides (LPS) and IL-4, respectively. Additionally, Jiang et al. (2014) reported the enhanced M2 activation along with a decrease in M1 activation in the context of mTORC1 deficient mice. However, in the current study, we found the simultaneous upregulation of M1- and M2-related genes. All of these differences with respect to M1 and M2 polarization may be due to the different experimental conditions, and especially, the different cells studied.

It should be emphasized that previous studies on M1/M2 polarization of Kupffer cells were generally based on the qPCR results of M1/M2 macrophage related markers, while our study showing the characterization of the expression of CD86 (M1 marker) and CD206 (M2 marker) in Kupffer cells via flow cytometry. Interestingly, in contrast to other tissue-resident macrophages in the body (Davies et al., 2013), Kupffer cells showed double positivity for the M1 and M2 markers. Since the Kupffer cells of control mice also showed the same feature, we believe that the CD86^+^CD206^+^ phenotype is likely a specific manifestation of Kupffer cells, instead of a result of macrophage-specific LAMTOR1 depletion. Therefore, the remarkable accumulation of M1 and M2 double-positive Kupffer cells resulted in the simultaneously induced expression of M1 and M2 macrophage-related genes in the liver. Interestingly, the phenotypic categorization of macrophages is currently under revision, since a wider spectrum of phenotypes has been discovered (Mosser and Edwards, 2008; Trombetta et al., 2018). Therefore, our data, particularly with respect to Kupffer cells, may contribute to the new categorization of macrophages.

We also observed that LAMTOR1-null macrophages were more prone to apoptosis. Several studies found that the LAMTOR1/mTORC1 pathway plays a crucial role in the late stages of lysosomal maturation (Takahashi et al., 2012). In fact, LAMTOR1 depletion strongly increased lysosomal catabolism leading to the production of excessive reactive oxygen species (ROS), which, in turn, triggered p53-dependent cell cycle arrest and apoptosis (Malek et al., 2012). Consequently, more monocyte-derived macrophages (MDMs) are recruited into the liver in order to maintain the barrier function of Kupffer cells, as our model clearly suggests.

Generally, notable inflammation results in fever. However, the HFD-fed LAMTOR1 MKO mice, with significantly elevated levels of pro-inflammatory cytokines, showed normal body temperature (Refinetti, 2010). Clinically, inflammation induced fever is usually triggered by hypothalamus, while the hyperinflammatory state induced by macrophage LAMTOR1 deficiency in this study is a congenital, non-infectious inflammation. Therefore, it seems that macrophage LAMTOR1 depletion may not affect the hypothalamic function.

This study is not without limitations. First, the underlying mechanism linking LAMTOR1 and inflammation is still unknown; therefore, further investigation is needed. Second, the liver is an organ composed of several types of cells, and in this study, we only focused on macrophages and hepatocytes. Therefore, the research must be extended to the other cell types, and the interaction between macrophages and the other hepatic cells must also be investigated.

Overall, our study shows that the macrophage-specific LAMTOR1 deficiency leads to the accumulation of Kupffer cells in the liver and to a hyper-inflammatory state that may improve systemic metabolism. Therefore, altogether, our results clearly suggest a role of Kupffer cells in hepatic lipid deposition, something that deserves to be investigated in the future.

## Data Availability Statement

The original contributions presented in the study are included in the article/Supplementary Material, further inquiries can be directed to the corresponding author/s.

## Ethics Statement

The animal study was reviewed and approved by the Ethics Committee of the Shanghai Jiao Tong University Affiliated Sixth People’s Hospital.

## Author Contributions

JY and YB designed the study. LY and MZ conducted the experiments and were the guarantors. LY performed the statistical analysis and wrote the manuscript. YS, XL, and JS participated in several related experiments. XM, JY, and YB revised the manuscript and contributed to the discussion. All authors contributed to the article and approved the submitted version.

## Conflict of Interest

The authors declare that the research was conducted in the absence of any commercial or financial relationships that could be construed as a potential conflict of interest.
