# Supplementary material for: Macrophage LAMTOR1 Deficiency Prevents Dietary Obesity and Insulin Resistance Through Inflammation-Induced Energy Expenditure
Source: Front Cell Dev Biol. 2021 May 20;9:672032. doi: 10.3389/fcell.2021.672032 (PMC8173123; doi:10.3389/fcell.2021.672032)
Supplement: Supplementary file 1 [file Table_1.DOCX]

Supplementary Table 1. Primers used in qPCR

| Target gene | Primers (5’ to 3’) |
| --- | --- |
| Acdc-f | CCCAAGGGAACTTGTGCAGGTTGGATG |
| Acdc-r | GTTGGTATCATGGTAGAGAAGAAAGCC |
| Ap2-f | AAGGTGAAGAGCATCATAACCCT |
| AP2-r | TCACGCCTTTCATAACACATTCC |
| Arg1-f | CTGAGCTTTGATGTCGACGG |
| Arg1-r | TCCTCTGCTGTCTTCCCAAG |
| β-actin-f | CGCTGCGCTGGTCGTC |
| β-actin-r | CCACGATGGAGGGGAATACAG |
| CCL2-f | GACCCCAAGAAGGAATGGGT |
| CCL2-r | ACCTTAGGGCAGATGCAGTT |
| CCL7-f | GGTCACGCCTAAGGAATGGTC |
| CCL7-r | ACATGAGGTCTCCAGAGCTTTAC |
| CD11b-f | AGCCAGCAGAGATGTTCAGT |
| CD11b-r | CCTTGGTTTTCACTGTCGCA |
| CD206-f | ATGGATTGCCCTGAACAGCA |
| CD206-r | TGTACCGCACCCTCCATCTA |
| Chi3l3-f | TGGAATTGGTGCCCCTACAA |
| Chi3l3-r | CCACGGCACCTCCTAAATTG |
| F4/80-f | TGAGTGCACCCAAGATCCAT |
| F4/80-r | TAACCAAGATCCCTGCCCTG |
| FAS-f | AGAGATCCCGAGACGCTTCT |
| FAS-r | GCCTGGTAGGCATTCTGTAGT |
| GAPDH-f | GGGTCCCAGCTTAGGTTCATC |
| GAPDH-r | ATCCGTTCACACCGACCTTC |
| HSL-f | TTCTCCAAAGCACCTAGCCAA |
| HSL-r | TGTGGAAAACTAAGGGCTTGTTG |
| IL-1β-f | CCACAGACCTTCCAGGAGAATG |
| IL-1β-r | GTGCAGTTCAGTGATCGTACAGG |
| IL-6-f | CTGGGGATGTCTGTAGCTCA |
| IL-6-r | CTGTGAAGTCTCCTCTCCGG |
| IL-10-f | ATAACTGCACCCACTTCCCA |
| IL-10-r | GGGCATCACTTCTACCAGGT |
| IL-12b-f | GTGGAATGGCGTCTCTGTCT |
| IL-12b-r | GAGGAACGCACCTTTCTGGT |
| Leptin-f | TGCTGCAGATAGCCAATGAC |
| Leptin-r | GAGTAGAGTGAGGCTTCCAGGA |
| LPL-f | GGACGGTAACGGGAATGTATG |
| LPL-r | ACGTTGTCTAGGGGGTACTTAAA |
| MRC1-f | AACAAAGGGACGTTTCGGTG |
| MRC1-r | TCCTTCTGCCCAATGTTTGC |
| NOS2-f | TCTTGGAGCGAGTTGTGGAT |
| NOS2-r | TGACACAAGGCCTCCAATCT |
| PPARγ-f | TGTGGGGATAAAGCATCAGGC |
| PPARγ-r | CCGGCAGTTAAGATCACACCTAT |
| RELMa-f | CAAGACACACCCCACCATCA |
| RELMa-r | CTCTATAGGAACTATGGATACTGCG |
| SREBP-f | AGTGGCAAAGGAGGCACTAC |
| SREBP-r | CTAGCTGGAAGTGACGGTGG |
| TNF-f | TGAGGTCAATCTGCCCAAGT |
| TNF-r | TGGACCCTGAGCCATAATCC |


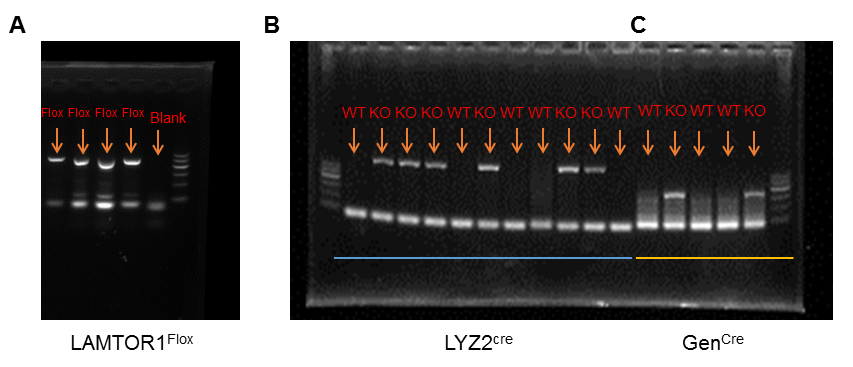


**Supplementary Fig. 1. LAMTOR1 MKO mice identification.** Identification results of (A) LAMTOR1^flox^, (B) LYZ2^cre^ and (C) Gen^cre^ primers measured by electrophoresis. Mice that positive for both LAMTOR1^flox^, LYZ2^cre^ and Gen^cre^ band were identified as LAMTRO1 MKO mice, that positive for LAMTOR1^flox^, but negative for LYZ2^cre^ and Gen^cre^ band were identified as WT mice.

Abbreviation: MKO: myeloid-specific knockout; WT: wild type.


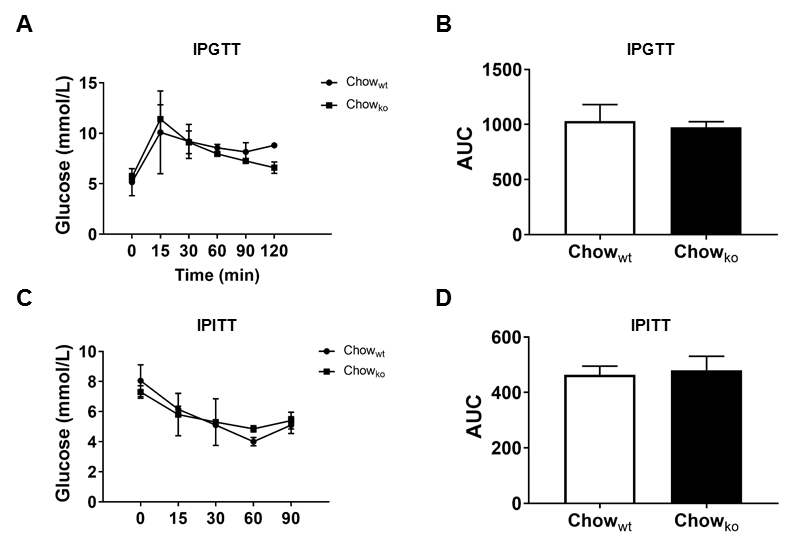


**Supplementary Fig. 2. Metabolic results in chow diet fed LAMTOR1 MKO mice and WT mice.** (A-B) **IP**GTT (glucose 1.5g/kg) and (C-D) IPITT (insulin 1U/kg) results. The data expressed as mean ± SEM, *n* = 3–5. ^*^, *P* < 0.05 *vs.* WT; ^**^, *P* < 0.01 *vs.* WT.

Abbreviation: IPGTT: Intraperitoneal glucose tolerance test; IPITT: Intraperitoneal insulin tolerance test; KO: myeloid-specific knockout; SEM: standard error of the mean; WT: wild type.

In terms of metabolic parameters, i.e., GTT and ITT, there was no significant difference between MKO and WT mice fed with chow diet.


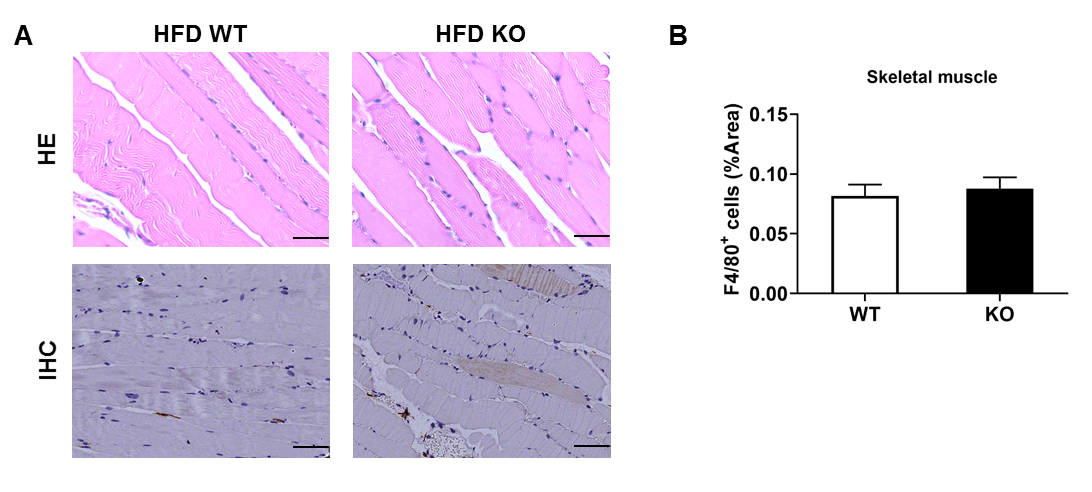


**Supplementary Fig. 3. Macrophage infiltration in skeletal muscle does not differ between HFD-fed LAMTRO1 MKO and WT mice.** (A) HE and F4/80 IHC staining and (B) IHC quantification of HFD-fed mice, presented as the percentage of area of F4/80^+^ cells in skeletal muscle; the data expressed as mean ± SEM, *n* = 4–6.

Abbreviation: HE: hematoxylin-eosin; HFD: high fat diet; IHC: immunohistochemistry; KO: myeloid-specific knockout; SEM: standard error of the mean; WT: wild type.

There are no differences in the general appearance, HE staining, or IHC staining (Supplementary Figure 3) in the skeletal (gastrocnemius and soleus) muscles from HFD-fed LAMTOR1 MKO and littermate control (WT) mice.


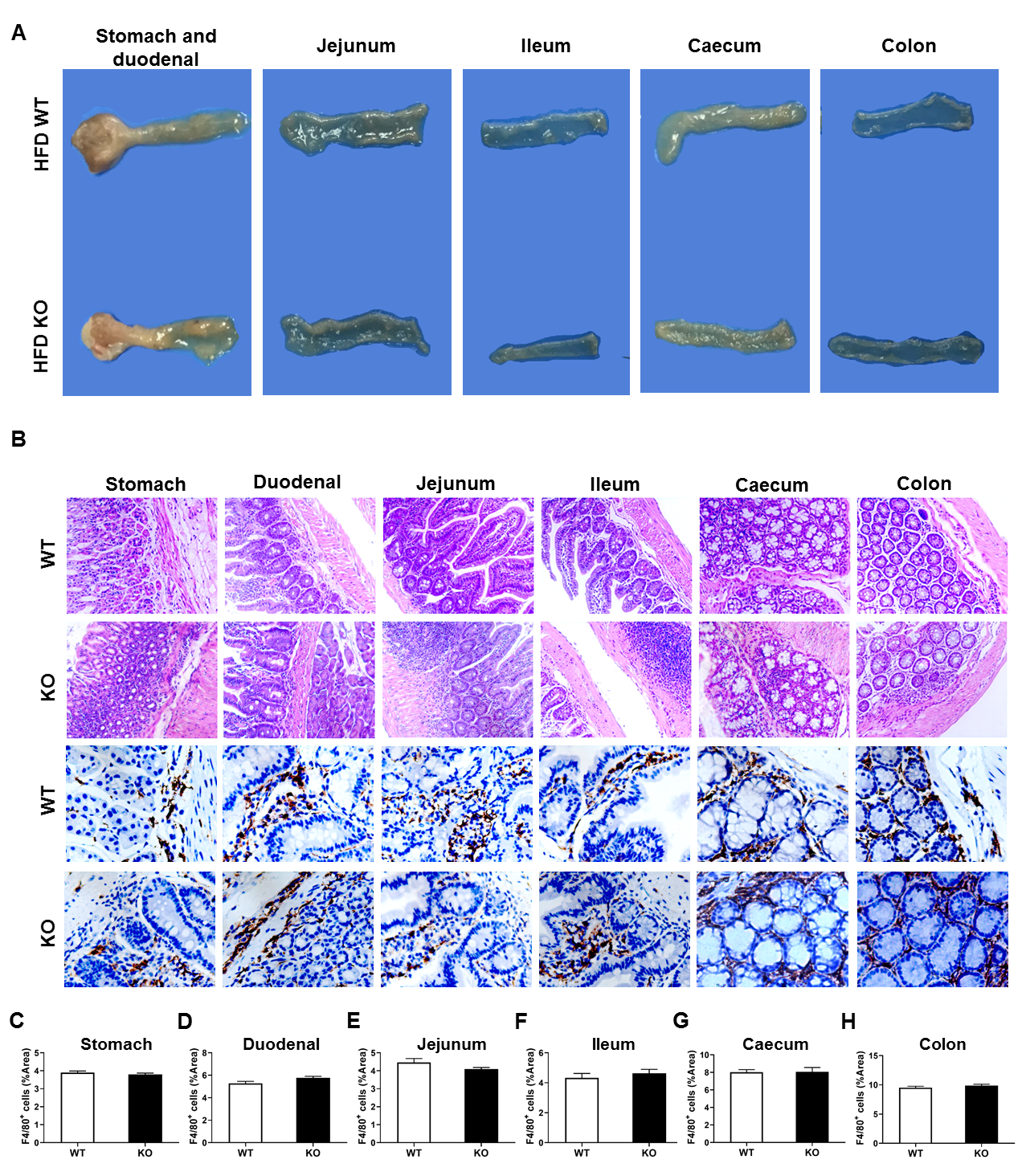


**Supplementary Fig. 4. Macrophage infiltration in gastrointestinal tract does not differ between LAMTOR1 MKO and WT mice when fed with HFD.** (A) Representative photos of Gastrointestinal tract of HFD-fed WT and LAMTOR1 MKO mice, along with its (B) HE and IHC staining (F4/80) results, and (C-H) IHC quantification of HFD-fed mice, presented as the percentage of area of F4/80^+^ cells in gastrointestinal tract. The data expressed as mean ± SEM, *n* = 4–6.

Abbreviation: HE: hematoxylin-eosin; HFD: high fat diet; IHC: immunohistochemistry; KO: myeloid-specific knockout; SEM: standard error of the mean; WT: wild type.

The general appearance of the gastrointestinal tract (including the stomach, duodenum, jejunum, ileum, cecum, and colon) of KO mice was normal. HE staining and F4/80 IHC staining showed no changes in the gastrointestinal mucosal structure and macrophage infiltration, respectively.


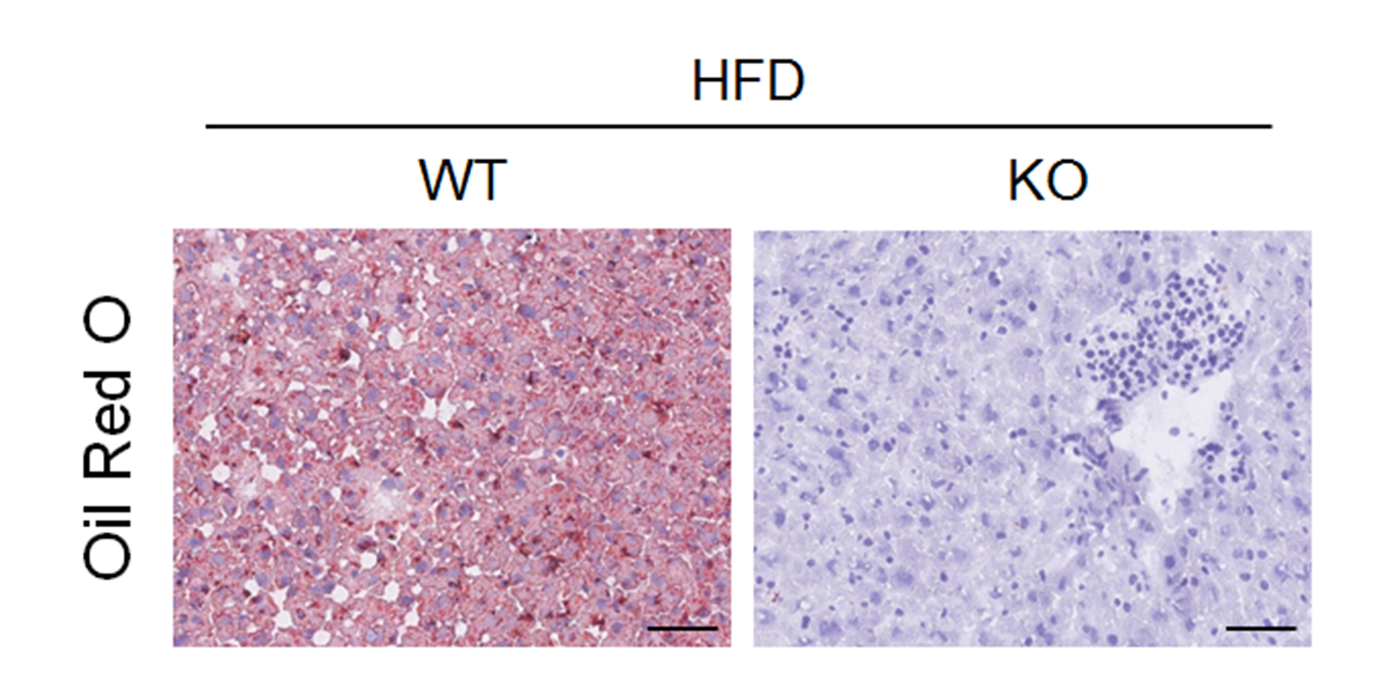


**Supplementary Figure 5. Oil red O staining in the liver of HFD-fed WT and KO mice.** *n* = 4–6. Abbreviations: HFD, high-fat diet; KO, myeloid-specific knockout; WT, wild-type.

When fed with HFD, the KO mice showed no hepatic steatosis in the oil red o staining.


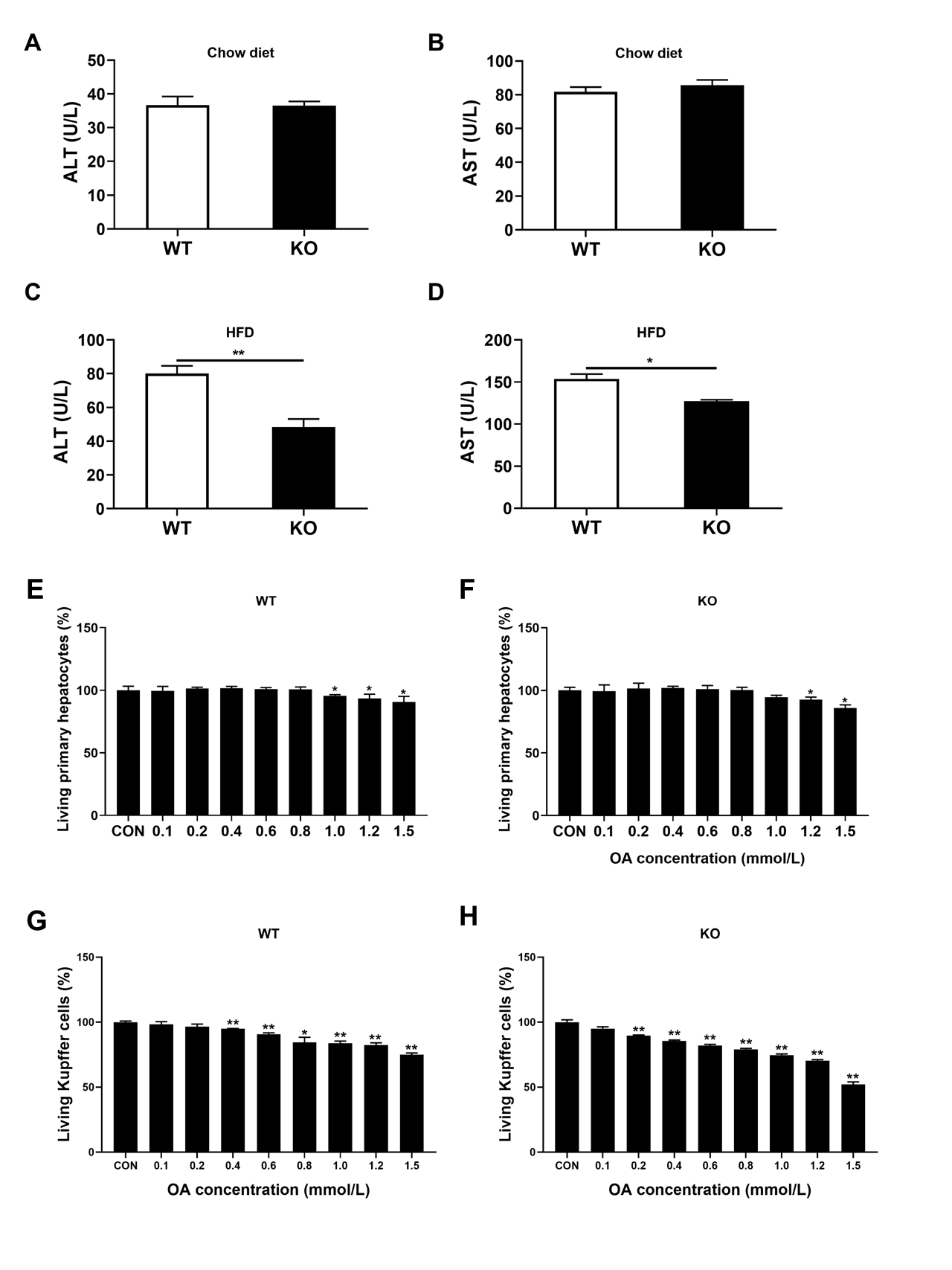


**Supplementary Fig. 6. The effect of HFD on liver function, as well as lipotoxicity of OA on primary hepatocytes and Kupffer cells.** (A-D) serum ALT and AST of chow diet and HFD-fed WT and KO mice; (E-H) The living cells of primary hepatocytes and Kupffer cells of WT and KO mice after treated with different concentration of OA for 24 hours. The cytotoxicity of OA was measured by MTT methods. The data expressed as mean ± SEM, *n* = 3–5.

Abbreviation: ALT: alanine aminotransferase; AST: aspartate aminotransferase; KO: myeloid-specific knockout; OA: oleic acid; SEM: standard error of the mean; WT: wild type.

Both ALT and AST showed no difference between chow diet-fed WT and KO mice. As for the HFD-fed mice, considering the improved hepatic lipid deposition, the KO mice showed a significantly lower ALT and AST levels compared with the WT mice (both *P* < 0.05). The MTT results demonstrate the dose effects of OA treatment on primary hepatocytes. As for the Kupffer cells, consistent with the results mentioned in the manuscript measured by CCK8, we found a decreased lipid tolerance in LAMTOR1 MKO mice.
